# NAS-HRIS: Automatic Design and Architecture Search of Neural Network for Semantic Segmentation in Remote Sensing Images

**DOI:** 10.3390/s20185292

**Published:** 2020-09-16

**Authors:** Mingwei Zhang, Weipeng Jing, Jingbo Lin, Nengzhen Fang, Wei Wei, Marcin Woźniak, Robertas Damaševičius

**Affiliations:** 1 College of Information and Computer Engineering, Northeast Forestry University, Harbin 150040, China; zhangmingwei98@nefu.edu.cn (M.Z.); linjingbo0618@nefu.edu.cn (J.L.); gaoithe@nefu.edu.cn (N.F.); 2 College of Computer Science and Engineering, Xi’an University of Technology, Xi’an 710048, China; weiwei@xaut.edu.cn; 3 Faculty of Applied Mathematics, Silesian University of Technology, 44-100 Gliwice, Poland; marcin.wozniak@polsl.pl (M.W.); robertas.damasevicius@vdu.lt (R.D.); 4 Department of Applied Informatics, Vytautas Magnus University, 44404 Kaunas, Lithuania

**Keywords:** deep learning, high-resolution remote sensing, image segmentation, neural architecture search, neural network optimisation, urban monitoring

## Abstract

The segmentation of high-resolution (HR) remote sensing images is very important in modern society, especially in the fields of industry, agriculture and urban modelling. Through the neural network, the machine can effectively and accurately extract the surface feature information. However, using the traditional deep learning methods requires plentiful efforts in order to find a robust architecture. In this paper, we introduce a neural network architecture search (NAS) method, called NAS-HRIS, which can automatically search neural network architecture on the dataset. The proposed method embeds a directed acyclic graph (DAG) into the search space and designs the differentiable searching process, which enables it to learn an end-to-end searching rule by using gradient descent optimization. It uses the Gumbel-Max trick to provide an efficient way when drawing samples from a non-continuous probability distribution, and it improves the efficiency of searching and reduces the memory consumption. Compared with other NAS, NAS-HRIS consumes less GPU memory without reducing the accuracy, which corresponds to a large amount of HR remote sensing imagery data. We have carried out experiments on the WHUBuilding dataset and achieved 90.44% MIoU. In order to fully demonstrate the feasibility of the method, we made a new urban Beijing Building dataset, and conducted experiments on satellite images and non-single source images, achieving better results than SegNet, U-Net and Deeplab v3+ models, while the computational complexity of our network architecture is much smaller.

## 1. Introduction

In recent years, with the progress and popularization of remote sensing technology, satellite imaging and aerial photography are becoming more and more advanced [1]. We can get images which contain large amounts of information. These images have been applied in many fields, like agriculture [2], forestry, geology, military, environmental protectio n [3], urban planning [4], etc. High-resolution (HR) remote sensing images include high spatial, temporal and spectral resolution. The HR remote sensing image in this paper mainly refers to the high spatial resolution (2 m resolution and better) remote sensing image. The high spatial resolution remote sensing images capture the surface of the earth in great detail. With the increasing spatial resolution of remote sensing images, there is a need to improve and innovate the method of analyzing remote sensing images.

Image segmentation of remote sensing images can be used for land area estimation, fire monitoring, urban planning, crop detection and yield modelling and many other applications [5,6]. Moreover, it is essential for observing the growth and evolution of complex urban systems, including slum detection, suburban growth, change in temperature in urban heat island, identifying disaster-damaged urban infrastructures, etc. [7,8,9,10].

Image segmentation aims to partition an image into homogenous regions such that no union of two adjacent regions is homogenous [11]. Remote sensing image segmentation has always been an important part of the remote sensing preprocessing process; how to improve the segmentation accuracy is always a difficult point [11]. Traditional HR remote sensing image segmentation is classified into four categories according to the principle of segmentation: the first is pixel-based algorithms, including the simplest segmentation algorithm—thresholding algorithm and cluster algorithm [12]. The second is boundary-based algorithms; the boundary-based algorithm usually looks for the sharp transition of gray value in the image to determine the boundary of the object region. The third is region-based algorithms, which are mainly divided into local regions based on the similarity between adjacent pixels to achieve segmentation. The fourth is physical-model-based algorithms, and the physical model is obtained from the imaging procedure, which describes the relationship between images and factual detail of the Earth’s surface [13].

With the popularization of remote sensing image segmentation and the development of artificial intelligence, the data-driven methods are getting more attention. Remote sensing image segmentation has attracted more attention from the computer vision and machine learning community. Convolutional Neural Networks (CNNs) have achieved state-of-the-art results in many computer vision tasks, which bring semantic segmentation into a new era [14,15,16]. As an improved architecture of CNNs, Fully Convolutional Network (FCN) demonstrated the state-of-the-art results for semantic image segmentation. FCN adopts deconvolution filter to conduct up-sampling on the feature map of the ultimate convolutional Layer. Compared to CNNs, FCN can recognize images at the pixel level and ensure robustness and accuracy simultaneously [17,18].

U-Net innovatively adopts the encoder–decoder architecture for semantic segmentation, i.e., the first half is divided into feature extraction and the second half is divided into upper sampling. U-net employs a totally different feature fusion method where features are spliced together in channel dimension to form a thicker feature [19,20]. Furthermore, in addition to the encoder–decoder structure, the fully connected Conditional Random Field (CRF), Atrous Convolution, Atrous Spatial Pyramid Pooling (ASPP), depth-separated convolution, and Xception technique are applied to the models in Deeplab family. This effectively improves the accuracy of boundary segmentation and the speed of training [21,22,23].

Although the above methods based on deep learning greatly enhance the accuracy and efficiency of remote sensing image segmentation [24,25,26], a robust model usually requires relevant experts to spend a lot of time and energy to complete it. Feature extraction and fusion are key for robust and effective image processing in remote sensing [27]. Especially due to the diversity of sources for remote sensing images, and the fact that the image features obtained by different methods are quite different [28,29], a method is required that can automatically search the optimal architecture for different data. The emergence of Neural Architecture Search (NAS) solves this pain point.

As an important derivative of automatic machine learning (Auto-ML), it replaces the manual process of architecture design as the machine’s automatic search for the neural architecture. MIT [30] and Google [31] proposed using reinforcement learning in 2016 to let computers automatically search for neural network architectures. The model obtained from the NAS achieves good accuracy in the image classification task. However, the initial neural architecture search required a large number of computing resources. For example, Google conducted an architecture search on the CIFAR-10 dataset, used 800 Graphical Processing Units (GPUs) and trained for 28 days. Such high computational costs make the work of ordinary researchers unrealistic. Therefore, how to reduce the cost of the search has become a problem that the NAS has had to face since its birth. Researchers have done a lot of work in recent years to get rid of high memory consumption [32,33].

Before doing an architectural search, we need to define the search space. The common search space is chained and it is formed by stacking lays with operators. Many deep neural networks have many similar parts, which are gradually abstracted into a cell, so the search space is greatly simplified. A cell is usually designed as a directed acyclic graph (DAG) [32,34,35,36].

There are three main types of search strategy. The first one is based on reinforcement learning. The generation of the architecture is regarded as an agent choosing the action, and the reward is obtained through the effect prediction function on a test set [30,31]. The second type of strategy is based on Genetic Algorithm (GA), a derivative-free optimization algorithm that may yield a global optimal solution, but is less efficient relatively [36,37]. The gradient-based method makes discrete search space continuous, and the objective function becomes a differentiable function, making it possible to use a gradient-based optimization method to find the optimal structure. The cell-based search space was applied into our works, and we use the gradient descent search strategy to search the space [32].

Here, we propose an improved HR remote sensing image segmentation method based on a neural architecture search, named NAS-HRIS. We applied NAS-HRIS to three different types of HR remote sensing dataset, to efficiently search out suitable architectures themselves.

**Figure 1:**
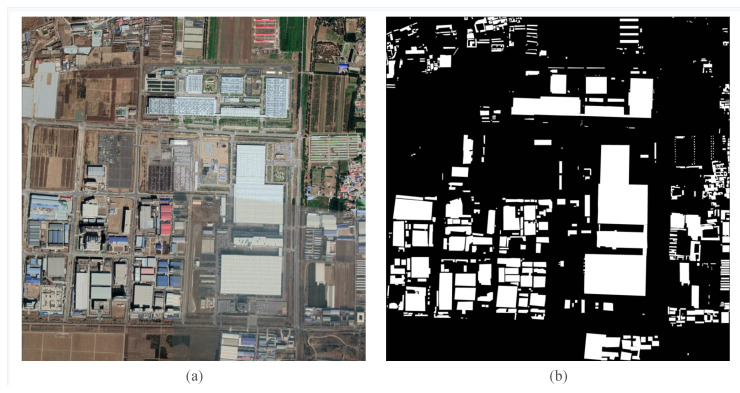
Example image from the Beijing building dataset (BBD), (**a**) is original data, and (**b**) is the associated label.

Summarizing, our contributions are listed as follows:

1. The NAS of the HR remote sensing image segmentation is explored for the first time;
2. Our work embeds DAG into the search space and designs the differentiable searching process, which enables learning an end-to-end searching rule by using gradient descent optimisation [38]. We use the Gumbel-Max trick to provide an efficient way to draw samples from a non-continuous probability distribution, and it improves the efficiency of searching and reduces the memory consumption;
3. We provide a new HR remote sensing image segmentation dataset: the Beijing building datasets (BBD) that can be useful for image segmentation applications such as building segmentation for urban planning; (Figure 1)
4. Conducted search on a variety of remote sensing images, and training was conducted in aerial images, satellite images and Google earth image, obtaining and we got 98.52% pix accuracy, and 90.44% Mean Intersection over Union (MIoU) by using NAS-HRIS on the WHU dataset.

Other parts of this paper are structured as follows. In Section 2, we provide our proposed methodologies in detail. The datasets, experimental settings and comparison results are presented in Section 3. At last, we discuss our work and put forward prospects for the future work in Section 4. We have released our code at https://github.com/zhangmingwei98/NAS-HRIS.

## 2. Methodology

**Figure 2:**
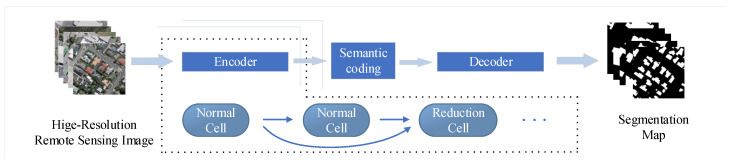
The model uses the encoder-decoder structure. And the encoder is composed of cells which are searched by the NAS.

In this article, we used NAS to construct the architecture of the encoder for the segmentation model Figure 2. The neural architecture search consists of three parts: search space design, search strategy formulation and evaluation method. We defined a search space composed of several cells, and we used the search strategy of gradient descent to select the weights of each edge of the directed acyclic graph, and so used the Gumbel-max trick to do continuous relaxation.

### 2.1. Architecture Search Space

#### 2.1.1. Cell Level

**Figure 3:**
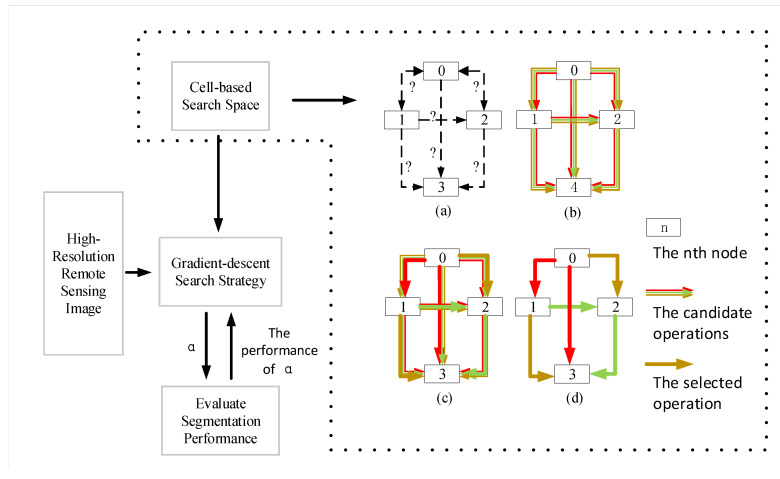
NAS-HRIS used the gradient descent search strategy to search the architecture of encoder in the cell-based search space and optimized the parameters by continuously evaluating the performance of the architecture. (**a**) operation of each edge in DAG is unknown; (**b**) candidate operations on each edge to continuous relaxation of the search space are set; (**c**,**d**) each edge is finalized by applying the reparameterization trick to sampling.

**Figure 4:**
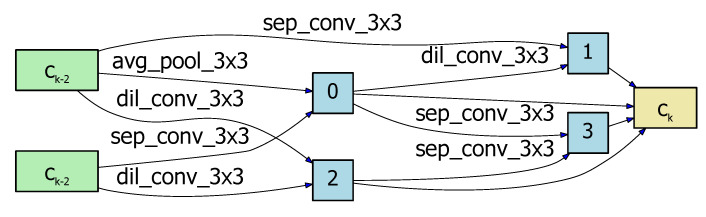
The normal cell searched by NAS-HRIS on WHUBuilding_Dataset.

**Figure 5:**
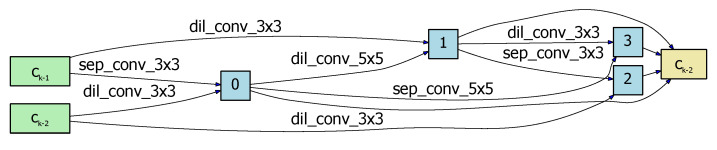
The reduction cell searched by NAS-HRIS on WHUBuilding_Dataset.

In NAS-HRIS, all cells are represented as a DAG (see Figure 3): the node of the graph stands for the input image or feature map, and the edge of the graph represents the operation, such as convolution and pooling. Each DAG consists of seven nodes: two are input nodes, four are intermediate nodes, and one is the output node. The output node is defined as the concatenation of four intermediate nodes as in Figure 4 and Figure 5. Our cell is designed according to [32,34,35,36]. The preorder node $n_{i}$ becomes the subsequent node $n_{j}$ after the calculation of operation *p* as follows

(1) $$N^{\left(j\right)}=\sum_{i<j}p_{i,j}\left(N^{\left(i\right)}\right)\;\;\;\;\;\;s.t.\;\;\;\;\;\;p_{i,j}∼P_{i,j}$$

In NAS-HRIS, the candidate operations set *P* has nine operations: (1) identity, (2) 3 × 3 avg pooling, (3) 3 × 3 max pooling, (4) 3 × 3 separate conv, (5) 5 × 5 separate conv, (6) 7 × 7 separate conv, (7) 3 × 3 dilated spearate conv, (8) 5 × 5 dilated spearate conv, (9) none.

#### 2.1.2. Network Level

We look for two different cells, i.e., a normal one and a reduction one. They are similar in structure, and their feature maps are padded. However, there is a difference between normal and reduction cells. The stride of all operations is set to 1 for the normal cell, whereas the stride is set to 2 for all operations at the reduction cell. The purpose of reduction cell is to reduce the feature map resolution.

In NAS-HRIS, a cell is treated as the basic block and stacked by certain rules to form neural network. We also apply DAG to structure the network topology. The two input nodes of cell $Cell_{k}$ are the output nodes of the preorder $Cell_{k-1}$ and $Cell_{k-2}$, respectively. Convolutions of 1×1 are filled in where necessary. In the network, reduction cells were set in the location of 1/3 and 2/3 of the total network depth. We define architecture variable as α and the weight of architecture as ω. α can be composed of $\alpha_{normal}$ and $\alpha_{reduction}$, $\alpha_{normal}$ and $\alpha_{reduction}$ are shared by all the normal and reduction cells, respectively. In our work, we search for $\alpha_{normal}$ and $\alpha_{reduction}$ values. NAS-HRIS selects the optimal operations from candidate operations according to the weight value in the search procedure. In the training procedure, we update the value of the selected operation by gradient descent.

### 2.2. Continuous Relaxation and Search Strategy

As we can see the search space in Figure 3, before the NAS-HRIS search architecture, the operation of each edge in DAG is unknown (a). We set up a certain number of candidate operations on each edge to continuous relaxation of the search space (b). Each edge of the finalized by applying the reparameterization trick to sampling (c,d).

Our goal is to gain the optimal architecture $\alpha^{*}$ and its weight $\omega^{*}$ within all operations. We introduced the loss function *L* to achieve our goal. $L_{train}$ and $L_{valid}$ are train loss and valid loss, respectively. We regard this problem as a bi-level optimization problem. We find $\alpha^{*}$ that minimizes $L_{valid}\left(\alpha^{*},\omega^{*}\right)$ in the case of obtaining the optimal weight $\omega_{\alpha }^{*}$, as we can see in (2) and (3).

(2) $$\min_{\alpha }L_{valid}\left(w_{\alpha }^{*},\alpha \right)$$

(3) $$s.t.\;\;\;\omega_{\alpha }^{*}=\arg\min_{\omega }L_{train}\left(\omega ,\alpha \right)$$

An architecture α consists of many repeating cells: $\lambda_{i,j}^{p}$ is the *p*-th element of a $\left|P\right|$-demensional learnable $\alpha_{i,j}$. We adopted the softmax function to get normalized probability $f_{i,j}^{p}$ for sampled operation *p* between $N_{i}$ and $N_{j}$. The process of selection a operation was relaxed, as can be seen in (4).

(4) $$f_{i,j}^{p}=\frac{\exp(\lambda_{i,j}^{p})}{\sum_{p^{\prime }\in P}\exp(\lambda_{i,j}^{p^{\prime }})}$$

In order to back-propagate gradient though $\lambda_{i,j}$, we propose using the Gumbel-Max trick [39,40] to re-formulate Equation (1), which makes it possible to sample from a discrete probability distribution in an efficient way, as can see in (5) and (6). This method is proposed to perform NAS for the first time in GDAS [41]. DARTS needs to keep all intermediate results in memory, but the Gumbel-Max trick selects only one operation at a time. Therefore, if there are *P* candidate operations, the computing resource consumption is about 1/P. Because the search efficiency of DARTS is mainly limited by memory resources, NAS-HRIS has a faster search speed in an environment with the same memory

(5) $$N_{j}=\sum_{i=1}^{j}\sum_{p\in P}\varphi_{i,j}^{p}p\left(N_{i};\omega_{i,j}^{p}\right)$$

(6) $$s.t.\;\;\;\;\;\;\varphi_{i,j}=\left\{\begin{array}{c} 1,\left(i,j\right)=\arg\max\left(\lambda_{i,j}^{p}+\varsigma_{p}\right)\\ 0,otherwise \end{array}\right.$$

where $\varsigma_{p}$ are Gumbel-distributed noise which are identically distributed and independently drawn samples from $G\mathrm{umbel}{\left(0,1\right)}^{1}$ in (7). The $\varphi_{i,j}$ vector we obtained is a one_hot vector, and we multiply this vector by the range vector of x, and we end up with the x that we’re sampling. $\omega_{i,j}^{p}$ is the weight of operation p∼P between $N_{i}$ and $N_{j}$.

(7) $$\varsigma_{p}=-\log\left(-\log\left(u\right)\right)\;\;with\;\;u\;∼Uniform\left[0,1\right]$$

We apply softmax to relax argmax in Equation (6), hence Equation (5) is differentiable. We replace $\varphi_{i,j}^{p}$ with approximately ${\tilde{\varphi }}_{i,j}^{p}$. This makes Equation (5) differentiable in back-propagation

(8) $${\tilde{\varphi }}_{i,j}^{p}=\frac{\exp(\left(\lambda_{i,j}^{p}+\varsigma_{p}\right)/\tau )}{\sum_{p^{\prime }\in P}\exp(\left(\lambda_{i,j}^{p^{\prime }}+\varsigma_{p^{\prime }}\right)/\tau )}$$

where τ is the softmax temperature.

NAS-HRIS use gradient descent to optimize $L_{valid}$, similar to using RL or evolutionary architecture search, where validation set performance is seen as reward or fitness. See Algorithm 1 for the detailed searching process, which uses the gradient descent method to fine-tune α and ω

(9) $$\omega =\omega -\xi \nabla_{\omega }L_{train}\left(\omega^{*},\alpha \right)$$

(10) $$\alpha =\alpha -\xi \nabla_{\alpha }L_{val}\left(\omega -\xi \nabla_{\omega }L_{train}\left(\omega ,\alpha \right),\alpha \right)$$

where ξ is learning rate.
**Algorithm 1** NAS-HRIS Search Encoder for High-Resolution Remote Sensing Image Segmention**Require:**$D_{train}$: the training set; $D_{valid}$: the validation set; *n*: batch size; initialized operation set *P*;**Ensure:**  1: initialized the architecture variable α and the weights ω randomly, learning rate ξ, search epochs  2: **repeat**  3:    Sample batch of data $D_{t}$ from $D_{train}$;  4:    compute $L_{train}\left(\omega ,\alpha \right)-D_{t}$;  5:    Updata ω by gradient descent:            $\omega =\omega -\xi \nabla_{\omega }L_{train}\left(\omega ,\alpha \right)$;  6:    Sample batch of data $D_{v}$ from $D_{valid}$;  7:    compute $L_{valid}\left(\omega ,\alpha \right)-D_{v}$;  8:    Updata ω by gradient descent:            $\alpha =\alpha -\xi \nabla_{\alpha }L_{valid}\left(\omega -\xi \nabla_{\omega }L_{train}\left(\omega ,\alpha \right),\alpha \right)$;  9: **until** converge

Compared with DARTS [32], NAS-HRIS saves $\left|P\right|$ times the GPU memory cost, making the implementation of NAS in large-scale datasets possible. This satisfies the large data characteristics of a high-resolution remote sensing image.

### 2.3. Evaluation criteria

There are many criteria to evaluate the segmentation effect, most of which are based on accuracy and IoU. And different criteria represent different evaluation meanings. We selected several representative indicators to represent the performance of the segmentation task. In order to easily represent these criteria, we set the number of positive samples correctly predicted as TP, the number of positive samples wrongly predicted as FP, the number of negative samples correctly predicted as TN, and the number of negative samples wrongly predicted as FN.

#### 2.3.1. Pixel Accuracy (PA)

This is one of the simplest metrics, and it represents the percentage of pixels that are properly classified.

(11) $$PA=\frac{TP+TN}{TP+TN+FP+FN}$$

#### 2.3.2. $F_{1}$ Score

$F_{1}$ Score is defined as the harmonic mean of the precision and recall.

(12) $$Precision=\frac{TP}{TP+FP}$$

(13) $$Recall=\frac{TP}{TP+FN}$$

(14) $$F_{1}=\frac{2\cdot Precision\cdot Recall}{Precision+Recall}$$

#### 2.3.3. Mean Intersection over Union (MIoU)

This is the standard metric for segmentation tasks. It represents the mean ratio of intersection to union of two sets.

(15) $$MIoU=\frac{TP}{TP+TN+FP}$$

## 3. Experiments and Results

We describe the implementation of NAS-HRIS on three different datasets in detail. All the experiments were done in a single Tesla V100 GPU which has 32G memory. Our experiments consist of three stages. First of all, we use NAS to search the optimal architecture on the specified dataset, according to Algorithm 1. After this step, we can get the certain normal cell and reduction cell. The second stage is to retrain the optimal architecture and obtain a better performance model. In the first two steps, the training set and validation set are used. At last, we use the testing set to assess the performance of the architecture we have searched. We define each cell as consisting of seven node and eight candidate operations, and the depth of the encoder is eight layers. The learning rate is 0.025.

### 3.1. Experiments on Aerial Dataset

We chose the WHUBuilding dataset [42] for aerial images. The dataset is composed of more than 22,000 independent buildings in Christchurch, New Zealand. These buildings are extracted from aerial images with a spatial resolution of 0.0075 m and a coverage area of 450 km${}^{2}$. Most of the images are down-sampled to 0.3 m spatial resolution and cropped into 8189 non-overlapping blocks to form the whole dataset. They are divided into three parts, 4736 images for training, 1036 images for validation, and 2416 images for testing.

**Figure 6:**
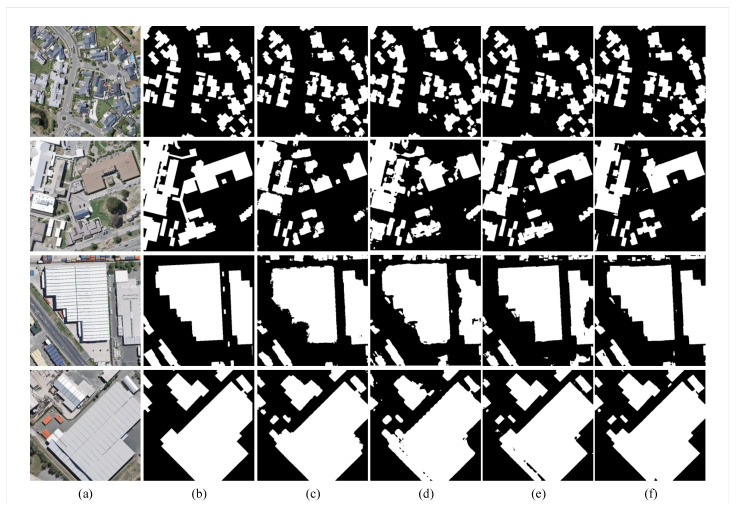
Examples of segmentation results with the SegNet, U-Net, Deeplab v3+ and NAS-HRIS, respectively, on the aerial dataset. (**a**) Image. (**b**) Label. (**c**) SegNet. (**d**) U-Net. (**e**) Deeplab v3+. (**f**) NAS-HRIS.

The architecture search process was carried out on the WHUBuilding dataset for about 12 hours for 30 epochs, and the resulting normal cell is shown in Figure 4, and the reduction cell in Figure 5. We ran the NAS-HRIS three times and the deviations of the PA, $F_{1}$, and MIoU were 0.12%, 0.38%, and 0.25%, respectively, indicating the MIoU being nearly invariant. We compared NAS-HRIS with SegNet, U-Net and Deeplab v3+. The comparison results are shown in Table 1 and Figure 6. As we can see, the MIoU was higher than 5.93% and the $F_{1}$ was higher, 4.81%, than SegNet. Due to the simple design of the search space, our model is very small, only 1/164 times the size of SegNet.

As can be seen in Figure 6, the ability of SegNet to divide independent buildings is strong, and there is little adhesion between buildings, but the integrity of building segmentation is not high. In the aerial HR remote sensing images, U-Net does not perform as well as in the field of medical images. Although the MIoU is higher than SegNet, the independence of segmentation is not strong, and it is difficult to distinguish the areas between buildings. In the three groups of control experiments, Deeplab v3+ is the most prominent; the edge of the building is clearly divided, but there will be regional misclassification in the middle part of the building. As can be seen from the third picture, the distinction between roads and houses is still a difficult point in building segmentation, especially in areas with similar features. Obviously, the best performance is NAS-HRIS, the edge is clear, and the building segmentation is complete.

We used search time and train time to measure our approach NAS-HRIS. Because Segnet, U-net and deeplab are fixed architecture, there is no search time, so we have listed the respective trian time in relevant experiments. It is worth mentioning that because the DARTS method consumes a lot of memory, especially in the case of high-resolution remote sensing images with such a large scale of data, experiments cannot run on 32G GPU, so we do not give the relevant data, which precisely reflects the significance of our method improvement.

### 3.2. Experiments on Satellite Dataset

**Figure 7:**
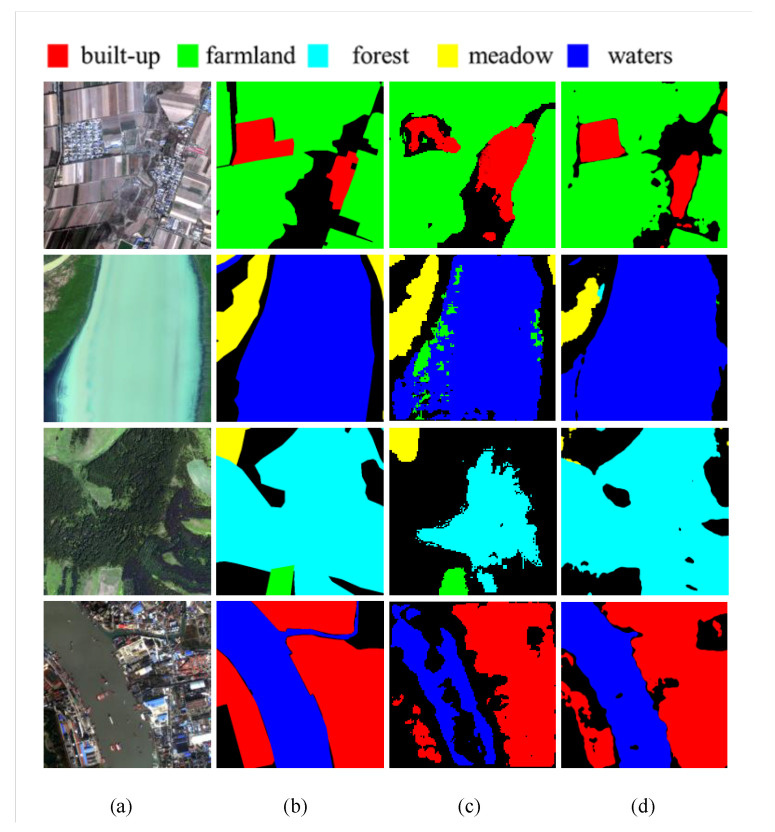
Representative cases of image segmentation results with SegNet and NAS-HRIS, respectively. (**a**) Image. (**b**) Label. (**c**) SegNet. (**d**) NAS-HRIS.

Gaofen Image Dataset (GID) is a dataset for land cover classification. It contains 150 HR images captured from more than 60 cities in China [43]. Each original image is 7200 × 6800, and we cut them into 182 images, each with a size of 512 × 512. Due to some problems with image labels, we selected 10,000 images as our dataset. Among them, 6000 images are for training, 2000 images for validation, and 2000 images for testing. There are five classes of tag in GID, which are built-up, farmland, forest, meadow, and waters, as can be seen in Figure 7.

By analogy with WHUBuilding, we used the three architectures of SegNet, U-Net, Deeplab v3+ as a comparison. The MIoU of NAS-HRIS is 7.37 % to 8.84 % higher than the other three methods (see Table 2), which shows the superiority of the customized architecture obtained by architecture search in complex datasets. Because there are many unmarked parts in the source image, in order to show the effect, we deliberately selected four images and compared them in this experiment. As can be seen from Figure 7, in satellite images, the two methods are not satisfactory for the boundary control of segmentation. There are functional disorders in the classification of forest by Segnet and functional disorders in the classification of meadow by NAS-HRIS. Note that in the last image, there are some ships parked on the water; although it is not marked in detail in the label, both methods have reflected that.

**Figure 8:**
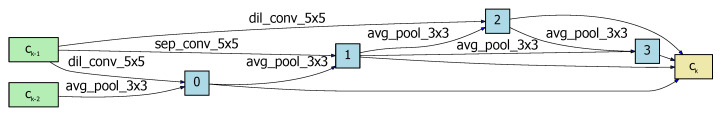
The normal cell searched by NAS-HRIS on GID_Dataset.

**Figure 9:**
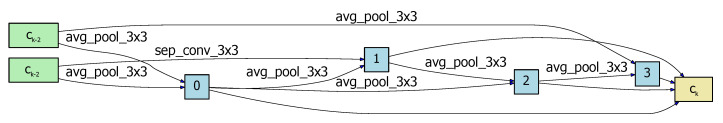
The reduction cell searched by NAS-HRIS on GID_Dataset.

Compared to the cells searched by NAS-HRIS on WHUBuilding_Dataset, the cells searched by NAS-HRIS on GID_Dataset in Figure 8 and Figure 9 have a large number of avg_pooling. The reason for our analysis is that GID_Dataset is a satellite image dataset, which has large area, many colors and complex features. Furthermore, avg_pooling retains more background information from a wide range of images.

### 3.3. Experiments on Non-single Source Dataset

In order to run NAS-HRIS in multiple environments, we have made a non-single source dataset, namely Beijing Building Dataset (BBD). It is worth mentioning that BBD not only meets the requirements of HR image segmentation labels, but also has the value of convenient application. BBD is an elevation satellite image dataset, which is integrated by satellite image and aerial photographs for building extraction and identification. It contains 2000 images from Google Earth History Map of five different areas in Beijing in November 24th, 2016, and all these images are 512 × 512 with a precision of 0.458 m. It covers more than 100 km${}^{2}$ geographic areas of Beijing both in suburbs and urban areas. We split the dataset into three parts, 1200 images for training, 400 images for validation and 400 images for testing.

**Figure 10:**
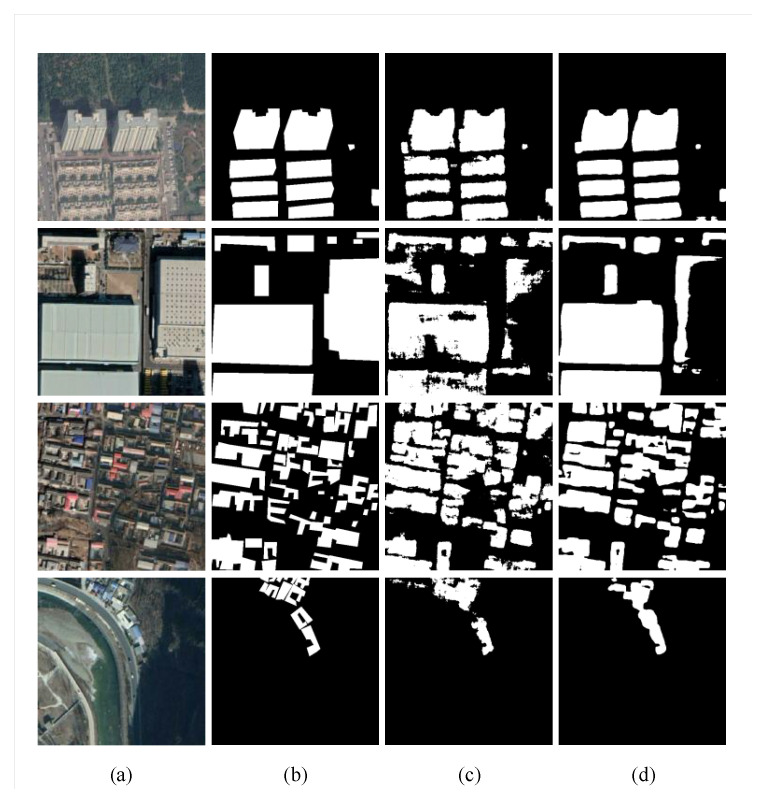
Examples of segmentation results with the SegNet and NAS-HRIS, respectively, on the non-single source dataset. (**a**) Image. (**b**) Label. (**c**) SegNet. (**d**) NAS-HRIS.

In this experiment, we used the architecture searched on the WHUBuilding datasets. On this basis, retrain was carried out. The results of NAS-HRIS compared with SegNet, U-Net and Deeplab v3+ are shown in Table 3 and Figure 10.

## 4. Discussions and Conclusions

We proposed an improved image segmentation algorithm for high-resolution (HR) remote sensing images based on a neural architecture search (NAS-HRIS). NAS-HRIS uses a gradient descent search strategy to search in a cell-based search space. Compared with traditional methods, NAS-HRIS realizes the automatic design of neural networks and reduces the memory resources used in the automatic search process. We created a new urban Beijing Building Dataset (BBD), which is an elevation satellite image dataset integrated by satellite image and aerial photograph for urban building extraction and identification. We applied NAS-HRIS to aerial images, satellite images, and non-single source images, and achieved 90.44% MIoU on the WHUBuilding dataset. Although NAS-HRIS performs well in the task of segmentation of the HR remote sensing datasets, it still needs to consume considerable computing resources in the process of searching the architecture. Therefore, in the following work, we will further optimize the search space and search strategy and get rid of the constraints of computing resources on the neural architecture search.

## Figures and Tables

**Table 1:** Testing result on WHUBuilding_Dataset

Architectures	Parameters (M)	PA (%)	$F_{1}$ (%)	MIoU (%)	Search Time (h)	Train Time (h)
SegNet	29.4441	97.77	88.96	84.51	-	7.4
U-Net	23.3565	98.30	93.56	88.41	-	6.2
Deeplab v3+	13.3953	98.09	94.47	90.20	-	4.0
NAS-HRIS	0.1868	98.52	93.77	90.44	12.1	16.4

**Table 2:** Testing result on GID_Dataset

Architectures	Parameters (M)	PA (%)	$F_{1}$ (%)	MIoU (%)	Search Time (h)	Train Time (h)
SegNet	29.4441	79.96	71.50	63.19	-	18.3 h
U-Net	23.3565	80.37	73.71	64.66	-	13.2 h
Deeplab v3+	13.3953	82.42	71.83	63.82	-	14.9 h
NAS-HRIS	0.1232	88.48	78.35	67.03	10.6	19.5 h

**Table 3:** Testing result on BBD_Dataset

Architectures	Parameters (M)	PA (%)	$F_{1}$ (%)	MIoU (%)	Search Time (h)	Train Time (h)
SegNet	29.4441	95.48	82.11	74.12	-	5.4
U-Net	23.3565	95.21	83.56	74.66	-	2.8
Deeplab v3+	13.3953	94.42	84.43	75.19	-	3.3
NAS-HRIS	0.2048	96.28	85.31	75.21	12.1	5.8
